# Supplementary figures and images for: A community-science approach identifies genetic variants associated with three color morphs in ball pythons (Python regius)
Source: PLoS One. 2022 Oct 19;17(10):e0276376. doi: 10.1371/journal.pone.0276376 (PMC9581371; doi:10.1371/journal.pone.0276376)

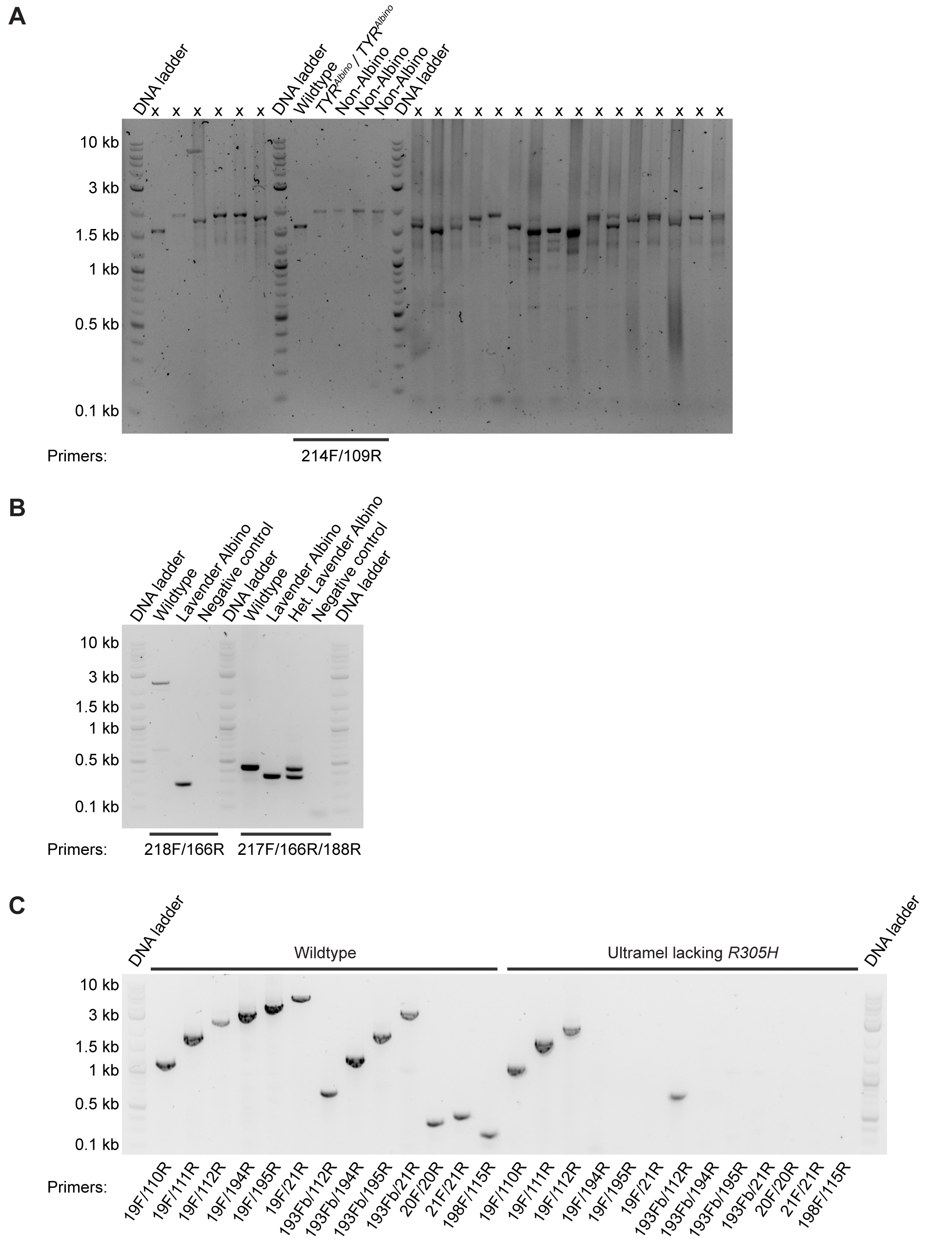

Supplement: S1 Fig — (A) Raw image of the gel displayed in Fig 2. (B) Raw image of the gel displayed in Fig 3. (C) Raw image of the gel displayed in Fig 4. Brightness and contrast settings have not been adjusted in these images. x, experiment unrelated to the current study. (TIF) [file pone.0276376.s001.tif]
