# Supplementary material for: A community-science approach identifies genetic variants associated with three color morphs in ball pythons (Python regius)
Source: PLoS One. 2022 Oct 19;17(10):e0276376. doi: 10.1371/journal.pone.0276376 (PMC9581371; doi:10.1371/journal.pone.0276376)
Supplement: Attachment — Submitted filename: Response to reviewers.docx [file pone.0276376.s008.docx]

**Responses to Reviewers**

*We thank the reviewers for their helpful comments in the manuscript. Their comments have improved the manuscript and strengthened the conclusions of our study.*

**Comments to the Author**

Reviewer #1: Brown et al. presents a thorough investigation into the genetics behind several ball python color morphologies (Albino, Lavender Albino, and Ultramel). Utilizing a community-science based, non-invasive approach to collecting DNA samples from shed skin, they were able to find putative loss-of-function mutations for each of the described color morphs. Provided their analysis was based on the highly fragmented, scaffold level assembly of the Burmese python, the authors did an nice job validating each of the gene annotations with other existing sources. Their analysis of each color morph was detailed, organized, and thoroughly explained results even when a clear answer was not found. This was evident by the multiple approaches used to attempt to identify a putative causal variant for the TYR-albino haplotype. Although they were unable to identify a specific causal variant, their analysis was thorough and convincing to show there was no clear answer within the scope of this study.

In all, I feel that Brown et al. made convincing arguments for all color morphologies described and their claims are supported by empirical data, statistical analyses, and previous literature. Given a portion of the study was conducted by undergraduates, they implemented stringent controls and checks throughout the analysis to ensure the data was up to standard and could be confidently included into the study. Their discussion and methods are well written and easy to follow, but the discussion may benefit from a few comments on how the fragmented Burmese python reference may have impacted their ability to accurately assembly genes fragmented across several scaffolds and the subsequent identification of causal variants from those fragmented genes. Especially with the identification of a variable number tandem repeat within a discontinuous region of the Burmese python assembly. Beyond the few comments below, Brown et al. make compelling arguments for the first analysis of multiple color morphologies in ball pythons.

*Thanks for this suggestion. We have added a paragraph to the Discussion section describing the impact of the fragmented Burmese python reference genome on our study. Main impacts of the fragmented Burmese python reference genome included (i) inability to interrogate TYR intron 1 in the TYR^Albino^ allele, and (ii) inability to the map the breakpoints of the putative deletion in TYRP1. The variable number tandem repeat was not affected by the fragmentation of the reference genome because this repeat resides in a region of the reference genome that was not fragmented. Our new paragraph is provided here:*

*"One challenge for genetic studies in ball python is the absence of a reference genome. To fill this gap, we relied on the genome of Burmese python [90], which is a scaffold-level assembly. Contigs in this assembly are small, and genes are sometimes fragmented across scaffolds. This fragmentation limited our analysis of the TYR^Albino^ haplotype and the putative deletion in TYRP1. For TYR^Albino^, we were unable to assess whether the TYR^Albino^ haplotype contained a large indel or rearrangement in the first intron of TYR, due to a discontinuity in the Burmese python genome in this region. For the putative deletion in TYRP1, we were unable to map the exact breakpoints of the deletion, due to a discontinuity in the Burmese python genome immediately downstream of TYRP1. As genomic resources for Burmese python and ball python expand, we expect to identify a putatively causative variant on the TYR^Albino^ haplotype and to map the breakpoints of the putative deletion in TYRP1. This information will increase the prospects for genetic testing for these alleles."*

*In response to the concern about gene annotations, we have updated the Methods section to more clearly describe the conservation of gene structure across species. Gene structure for the genes analyzed in this study were highly conserved. Genes contained the same number of exons in all species, and the intron-exon boundaries were perfectly conserved across species (with the exception of one slight difference of one boundary for one gene in mouse). Moreover, every coding region of every gene was present in the Burmese python reference assembly. It is true that some genes were split across scaffolds, but these splits always occurred in introns. Thus, while it remains theoretically possible that our new gene annotations contain minor errors, our confidence in these gene annotations is high. Our new passage is provided here:*

*"A main cause of these errors was that the Burmese python genome (Python_molurus_bivittatus-5.0.2) is a scaffold-level assembly; many genes were split across scaffolds. We therefore curated new gene annotations in Burmese python, using conservation of gene structure across species. Alignment of protein sequences for each gene from corn snake, anole lizard, chicken, and/or mouse revealed that gene structure was highly conserved for all seven genes: Genes in each species contained the same number of coding regions, and the coding-region boundaries relative to protein sequences were perfectly conserved across species, with the exception of one slight difference in mouse for one boundary of SLC45A2 (S2 Table). We therefore felt confident that conservation of coding-region boundaries could be used to curate new gene annotations in Burmese python. While it remains theoretically possible that our new gene annotations contain minor errors, our confidence in these gene annotations is high, given the high conservation of gene structure across species."*

On page 8, line 142 it says "These results demonstrate and association between…" I'm assuming they meant "demonstrated an association".

*Thank you for finding this typo. We have corrected it.*

On page 9, line 148 it says " We found that one of these animala was homozygous…" I'm assuming they meant "animals".

*Thank you for finding this typo. We have corrected it.*

On page 13, line 254 it states "We found that all four Lavender Albinos were homozygous for the deletion (Figure 3C-D)". However, Figure 3D shows there are 5 homozygous Lavender Albinos and the legend also states there are 5. Please clarify how many Lavender Albinos were used.

*This table includes the original Lavender Albino animal in which the OCA2 deletion was identified. We have updated the figure caption to indicate the inclusion of this animal. The same is true for the Ultramel table in Figure 4. We have updated the Figure 4 caption in the same way.*

Reviewer #2: This study investigates the genetic underpinnings of three color mutations in a common pet snake, the ball python, Python regius. Using known loss-of-function mutations across a variety of vertebrate species, the authors identified a suite of genes from which the Burmese python genome could be used for primer design. Three mutations were identified corresponding to the albino mutation, one for the lavender albino variant, and both a mutation and a deletion were found in the ultramel variant. The study is novel, and the student center approach is commendable. My concerns are minor.

1) The sample sizes for the color variants are very low. E.g., 20 albino, 5 albino/candy, 1 albino/toffee, and 1 toffee; 5 lavender albinos, 3 ultramels. As such. The confidence in identifying the causal mutations may also be low.

*We have increased the sample size for each morph. The Albino/Toffee/Candy sample was increased from 27 to 50. The Lavender Albino sample was increased from 5 to 14. The Ultramel sample was increased from 3 to 11. These expanded sample sizes support the conclusions in the original manuscript.*

*We recognize that larger samples would be preferable. However, obtaining additional samples was not possible. We have collected over 1,400 samples from owners and breeders across the US. Our small sample sizes reflect the rareness of the Albino/Toffee/Candy, Lavender Albino, and Ultramel morphs.*

*We cannot exclude the possibility that additional alleles exist for the morphs described in this manuscript. However, such alleles are necessarily rare, given their absence in our sample. We have amended out Discussion section to include the following statement, "Due to the small sample size of our study, we cannot exclude the possibility of additional loss-of-function alleles of these genes. However, such alleles are expected to be at low frequency in the ball python population, given their absence from our sample."*

2) The definition of unrelated is relatively weak. Obtaining samples from different breeders does not make the animals unrelated. In fact, many of the breeders listed as assisting commonly source animals from one or just a few other breeders. Therefore, there is likely a lot of shared recent ancestry. The authors should therefore consider generating a more thorough pedigree for each sample. Basically, to which origin can the variants be traced back to.

*This argument is a fair point. We have removed the word ‘unrelated’ from the manuscript. We have added a description to the Methods Section justifying our decision to limit our sample to animals contributed by different contributors. This description indicates that our sampling strategy was designed to increase genetic diversity within each morph. Our description also indicates that although we cannot exclude that some animals in our sample might be close relatives, our discovery of multiple alleles for two morphs (Albino and Ultramel) indicates that our samples include animals from different lineages. In addition, our description indicates that generating a thorough pedigree for each animal was not possible because most owners lacked this information. Our revised passage is provided below:*

*“To maximize genetic diversity within each category of morph, we limited our sample of animals of any one morph to animals contributed by different contributors.* *Exceptions were made if animals contributed by the same contributor had been obtained from different breeders. The goal of this strategy was to reduce the number of animals that were close relatives (e.g. siblings or parent-offspring pairs). Obtaining full pedigree information was not possible because most owners lacked this information (e.g. “I bought my animal at a pet store” or “I got my animal from a friend who was moving away”). Our discovery of multiple alleles for the Albino and Ultramel color morphs supports the idea that animals in our sample were derived from multiple lineages. However, we cannot exclude the possibility that some animals in our sample may have been close relatives.”*

3) I feel that the sample size for the ultramel variant is incredibly low. Given both the convoluted history of that variant (at least three imported animals over the space of 10 to 15 years), and uncertainty that these animals labelled as “ultramel” are compatible (i.e., will produce an “ultramel” variant if crossed), means that the result is a little speculative. I suppose it could be framed as “animals labelled by a breeder as ultramel exhibited two distinct alleles”, not “the ultramel trait is underpinned by two alleles”. The reason I say this is that one of these alleles might correspond to one of the previously important animals with a similar phenotype, and over time, that has been lumped in as being an “ultramel”. The authors should therefore maybe consider either 1) obtaining more samples of “ultramel” animals, or 2) obtaining samples from the other lines with a similar trait (e.g., the monarch trait and Crider trait). It might simply be case of mislabelling (known or unknown) by the breeder.

*We have increased our sample size of Ultramels from 3 to 11. These 11 animals included six animals homozygous for the TYRP1 R305H missense variant, three animals homozygous for the TYRP1 putative deletion, and two compound heterozygotes. Thus, this larger sample size supports the conclusion that animals described as Ultramel are a mixture of multiple genotypes, but are always homozygous or compound heterozygous for loss-of-function alleles of TYRP1.*

*A major difficulty in studying the genetics of ball pythons is that the origins of ball python morphs are not well documented in the scientific literature. We have heard multiple stories about the origins of Ultramel morph, but none of these stories have been officially documented. Likewise, we have heard multiple thoughts about whether Ultramel is allelic with morphs having similar coloration (e.g. Monarch, Crider). We have attempted to obtain samples from these other morphs, but breeders have been unwilling to send samples. (In some cases, breeders have shared with us that they are purposefully withholding samples out of concern that our discoveries might decrease the value of their animals). Thus, it remains possible that one or more of these morphs is allelic with Ultramel. This possibility does not conflict with our conclusions that the Ultramel morph is caused by multiple loss-of-function alleles of TYRP1.*

4) I am disappointed that the authors did not include animals heterozygous for these traits, which are all readily available in collections. This would help confirm the diagnostic nature of the study.

*We purposefully excluded heterozygotes from our study because we have found that animals described as heterozygous for recessive morphs are not always heterozygous for these morphs. This situation likely arises from mislabeling by owners and breeders (intentional or non-intentional), perhaps in some cases from mis-attribution of paternity. We have added passages to the Methods Section justifying our exclusion of animals described as heterozygotes. The relevant passage is below:*

*“The total set of animals comprised 50 Albinos, 14 Lavender Albinos, 11 Ultramels, one animal described as heterozygous for Lavender Albino, and 46 animals described as having normal coloration (i.e. wildtype) or belonging to color morphs other than Albino, Lavender Albino, or Ultramel. Phenotypes of Albinos, Lavender Albinos, and Ultramels were confirmed by examining shed skins for a reduction of brown-to-black coloration. The single animal described as heterozygous for Lavender Albino was included to demonstrate the three-primer assay for genotyping the OCA2 deletion. Heterozygotes for Albino or Ultramel and additional heterozygous for Lavender Albino were excluded from our study because we have found that animals described as heterozygous for recessive traits by breeders and owners are sometimes not heterozygous for these traits (e.g. due to mis-attribution of paternity).”*

That said, my concerns are relatively minor and the study identifies genes and putative alleles for these three traits.

*Thanks for the positive feedback.*

Minor comments:

Ln 158 – The different albino variants needs to be identified here. I recommend using pictures. Toffee and Candy variants are phenotypically identical but are from different origins. I agree with the conclusion that they are the same variant. This is likely the case with many color and pattern variants in this species.

*Thanks for this suggestion. We have updated Figures 2 and 5 to include images of Candy and Toffee animals.*

Ln 383 – Caramel albinos are not necessarily infertile, although a reduced fertility rate has been observed. The main issues was that with some lines of caramel albino (they have been imported several times form the wild), the offspring exhibited severe spinal kinking, and hence non-viable.

*Thanks for the additional information about Caramel Albinos. We have updated the passage about Caramel Albinos to indicate that multiple lines of this morph have been identified and that defects have included spinal kinking and reduced female fertility. Our updated passage is included here:*

*“Our finding of multiple TYRP1 alleles for the Ultramel color morph was unexpected. This morph was not previously described as having multiple alleles. We speculate that one of the two alleles associated with the Ultramel color morph may represent an allele originally associated with a morph known as Caramel Albino. Multiple lineages of Caramel Albinos have been described, and their coloration similar to Ultramels. Caramel Albinos have been disfavored among owners and breeders because of spinal kinking and reduced female fertility. Caramel Albinos were not included in our study because we were unable to recruit any Caramel Albino samples. We speculate that the Caramel Albino morph may be allelic with Ultramel, and that one of two TYRP1 alleles associated with Ultramel may represent an allele originally described as Caramel Albino. Alternately, Ultramel may be distinct from Caramel Albino, and the two TYRP1 alleles may represent two distinct origins of the Ultramel morph.”*
