# Supplementary material for: A community-science approach identifies genetic variants associated with three color morphs in ball pythons (Python regius)
Source: PLoS One. 2022 Oct 19;17(10):e0276376. doi: 10.1371/journal.pone.0276376 (PMC9581371; doi:10.1371/journal.pone.0276376)
Supplement: Attachment — Submitted filename: Response to reviewers v3.docx [file pone.0276376.s009.docx]

**Responses to Reviewers**

*We thank the reviewers for helping to identify that our data had not been made publicly available in GenBank. We have remedied the problem, which strengthens the utility of our study.*

**Comments to the Author**

Reviewer #1: The authors have provided a revised version of their manuscript, answering all of my comments as well as the other reviewers comments in a manner that in my opinion satisfies the changes required for acceptance. The only minor revision that remains is the availability of their sequence data on GenBank. The authors provide GenBank accession numbers (MZ269492-MZ269502), but the sequences do not appear to be publicly available yet as no results are returned when queried. Once the data is fully available and accounted for, I feel the manuscript is ready to proceed to publication.

*Thanks for noticing that GenBank had failed to release the data associated with our publication. We have contacted GenBank and fixed the problem. The sequences associated with accession numbers MZ269492-MZ269502 are now publicly available. Thus, our publication now meets the data availability policy of PLoS ONE.*
