# Supplementary material for: A community-science approach identifies genetic variants associated with three color morphs in ball pythons (Python regius)
Source: PLoS One. 2022 Oct 19;17(10):e0276376. doi: 10.1371/journal.pone.0276376 (PMC9581371; doi:10.1371/journal.pone.0276376)
Supplement: Attachment — Submitted filename: Response to reviewers v4.docx [file pone.0276376.s010.docx]

**Responses to Reviewers**

We thank the reviewer for their helpful comments on the manuscript. These comments have improved the clarity of writing and will help readers navigate our manuscript.

Reviewer #3: The authors describe putative variants in three genes associated with different color morphs of the ball python. The study is well described, methodology detailed, and overall interesting for the field. It shows another example of a succesful collaboration between public and researchers using the citizen science approach. I also find it very cool that the authors include and acknowledge the undergraduate students contribution. However, I found a few ambiguities and formal genetic issues that should be corrected before publication.

Fig. 1 is the first place talking about "Candy" and "Toffee". These should be mentioned and explained in the introduction (around L86/87). Are they variations of "Albino"? From looking at this Figure, they seem to be visually closer to the "Ultramel" as they both seem to have darker and more brownish coloration between the yellow spots, compared to the white "Albino" or kind of cream in "Lavender Albino". Why are they considered albino? This needs to be explained in the introduction section. -edit: I have found the relevant section in L218-224. That is way too late, please move that paragraph into Introduction.

Thank you for pointing out this lack of clarity regarding the phenotypic variability within the Albino color morph. We have addressed this issue in two ways. First, we have added a sentence to the caption of Figure 1 explaining that the Albino color morph is phenotypically variable. This sentence reads:

“Phenotypes within the Albino color morph are variable, with some animals having skin patches that are white and others having skin patches that are light beige.”

Second, we have modified the text introducing the Albino color morph to explain the phenotypic variability within the morph. This paragraph reads:

“The Albino color morph in ball pythons is characterized by an absence or near absence of melanin in the skin and eyes—the brown-to-black coloration observed in wildtype is absent or severely reduced, and skin patches appear white or light beige (Fig 1D-F). The Albino color morph is described by breeders as having three alleles (AlbAlbino, AlbCandy, and AlbToffee), although prior to the current study, it remained unclear whether these alleles represented distinct molecular variants of the same gene or the same molecular variant discovered independently three times. We began our analysis of the Albino color morph by treating all animals within the color morph as a single group.”

Table 1 lacks some identified species, e.g. multiple TYRP1 variants in dog are well known (<https://www.omia.org/OMIA001249/>). Also, the dog reference (78) is incorrectly assigned to SLC24A5, instead of SLC45A2, where it is missing (<https://www.omia.org/OMIA001821/9615/>). I suggest the authors use the the recent comprehensive review for the canine variants (10.1111/age.13154). It would be also desirable to link the table gene entries to OMIA database for full reference.

Thank you for helping us improve this table. We have added a reference to the suggested review article describing color phenotypes in dogs. We have also fixed the error for the reference of SLC45A2 in dogs. Lastly, we have added OMIA links for the five genes with OMIA pages.

L116 "Similar phenotypes occur in other animals, where the loss of melanin extends to feathers and scales" should also include fur.

We have revised the text accordingly. Thank you for the suggestion.

L139 What is "Non-Albino"? In the Methods section, these are not termed. I presume those are the "...normal coloration (i.e. wildtype) or belonging to color morphs...", so just define the term in Methods (L489).

We have added a text to the Methods section to define this term. This text reads,

“We use the term Non-Albino to refer to animals having normal coloration or belonging to morphs other than Albino.”

Please clarify if there are 46 or 56 of these "Non-Albino" individuals. Fig. 2 and the paragraph above states 56 as opposed to 46 stated in the Methods L488 and labeled "Non" in the first column of Table S6. If you used other colors as controls, that needs to be clearly specified.

We have clarified this point as follows,

“The Non-Albinos included some Lavender Albinos and some Ultramels.”

Fig. 2C should have title changed from "TYR genotype" to "TYR haplotype".

We respectfully disagree. This portion of the figure shows diploid combinations of haplotypes, which constitute genotypes. Thus, the term genotype is the more appropriate term here.

L152 Include some numbers here and refer to Fig. 2C. How many homozygotes and comp. heterozygotes? It should be clearly stated (without the need of opening supplementary file) that the non-albino animals were not found homozygous for the albino-associated haplotypes.

Thank you for pointing out this lack of detail. We have revised the test accordingly. It now reads,

“The association for TYR was driven by two features of the haplotype distribution: (i) haplotype diversity was reduced from a total of 15 TYR haplotypes among Non-Albinos to three haplotypes among Albinos (Fig 2A, C), and (ii) two of the three TYR haplotypes found in Albinos were rare among Non-Albinos (Fig 2A). These results demonstrate an association between the Albino color morph and variants in TYR; further, they show that all Albino animals were homozygous (34 animals) or compound heterozygous (16 animals) for any of three haplotypes of TYR.”

Stating here that Non-Albinos were not found to be homozygous for the Albino-associated haplotypes is misleading because one of the Albino-associated haplotypes is actually quite common among Non-Albinos (Fig 2A). The reason is that this haplotype exists in two versions within the population. One version contains a missense variant that causes the Albino color morph (P384L). The other version does not contain this missense variant. At this point in the text, we have not discovered the missense variant and cannot distinguish between the two versions of the haplotype. Once we discover the missense variant (in the paragraphs immediately following the paragraph in question), we state that Non-Albinos were never found to be homozygous for the missense-containing version of the haplotype. We have revised this text to make it more it clear. The text reads,

“We hypothesized that D394G, P384L, and an unidentified variant on the TYRAlbino haplotype were causative for the Albino color morph. This hypothesis predicted that all Albinos would be homozygous or compound heterozygous for these variants, whereas Non-Albinos would be heterozygotes and non-carriers. To test this prediction, we genotyped our full panel of 50 Albinos and 56 Non-Albinos for the missense variants D394G and P384L. Consistent with our prediction, we found that the Albinos were exclusively homozygous or compound heterozygous for D394G, P384L, or the TYRAlbino haplotype (Fig 2C). The Non-Albinos were exclusively heterozygotes or non-carriers (Fig 2C). .”

L167 (and throughout for all others) Define the variants using the correct nomenclature standard (<https://varnomen.hgvs.org/recommendations/protein/variant/substitution/>), at least for the first time mentioned. E.g. the D394G should read UPQ41330.1:p.(Asp394Gly). Additionally, this describes a protein variant; therefore, it cannot be "in the coding region of a gene" - please, rephrase the sentences accordingly. As an example, this sentence should read: "We found that one of these animals was homozygous for a missense variant in the third exon of TYR (MZ269492:c.A3695G), which leads to an aspartic acid-to-glycine exchange (UPQ41330.1:p.(Asp394Gly); termed hereafter D394G)."

Thank you for this suggestion. We have revised the text accordingly. It reads,

“We found that one of these animals was homozygous for a missense variant in the third coding region of TYR (MZ269492:c.A3695G), which leads to an aspartic acid-to-glycine exchange [UPQ41330.1:p.(Asp394Gly)]. This variant is termed hereafter D394G. A second animal was homozygous for a different missense variant, also in the third coding region of TYR (MZ269492:c.C3665T), which leads to a proline-to-leucine exchange [UPQ41330.1:p.(Pro384Gly)]. This variant is termed hereafter P384L. .“

L239-248 The first paragraph is not about results. I suggest moving the part between L242 and L246 into Discussion.

We respectively disagree that this paragraph should be moved to the Discussion. The purpose of this paragraph is to introduce the hypothesis that the Lavender Albino color morph is caused by loss of function of OCA2. We have rewritten this paragraph to make its purpose clearer,

“The Lavender Albino color morph is characterized by skin patches that are lavender instead of brown or black. This phenotype is thought to arise from melanin levels that are dramatically reduced but not entirely eliminated. We hypothesized that this phenotype was caused by loss of function of OCA2, which encodes a chloride channel required for maintaining the pH of melanosomes [88,95]. When the OCA2 protein is absent or non-functional, the enzymes that synthesize melanin are less active, and only small amounts of melanin are produced [96,97]. The resulting phenotype is typically intermediate in severity between the loss-of-function phenotypes of TYR versus other melanogenesis genes (Table 1). OCA2 was therefore a good candidate for the causative gene of the Lavender Albino color morph.”

L281-293 The first paragraph is not about results. I suggest moving the part between L284 and L291 into Discussion.

We respectively disagree that this paragraph should be moved to the Discussion. The purpose of this paragraph is to introduce the hypothesis that the Ultramel color morph is caused by loss of function of TYRP1, TYRP2, SLC7A11, SLC24A5, or SLC45A2. We have rewritten this paragraph to make its purpose clearer,

“The Ultramel color morph is characterized by skin patches that are tan or light brown, rather than dark brown or black. This phenotype suggests a mild loss of melanin. We hypothesized that this phenotype was caused by loss of function of one of five genes: TYRP1, TYRP2, SLC7A11, SLC24A5, and SLC45A2 (Table 1). TYRP1 and TYRP2 encode enzymes involved in synthesizing melanin [87]. Their loss-of-function phenotypes are mild because of partially redundancy with other enzymes in the melanin synthesis pathway. SLC7A11 encodes a transporter responsible for importing cystine into the cell [99]. Cystine is a precursor to some forms of melanin, and its reduction alters melanin levels [77]. SLC24A5 encodes a K+-dependent Na+-Ca2+ exchanger [89]. SLC45A2 encodes a putative sugar transporter [90]. Loss of their encoded proteins reduces melanin through mechanisms that may involve defects in regulation of melanosome pH [84,89,100]. These fives genes were therefore good candidates for the causative gene of the Ultramel color morph.”

L297 Same comment as above re nomenclature. The coding variant should be designated with bp position and the actual substitution. The amino acid exchange is the consequence of the exonic variant.

Thank you for this suggestion. We have revised the text accordingly. It reads,

“Comparison of these sequences to wildtype revealed a single coding variant: a missense variant in the fourth coding region of TYRP1 (MZ269497:c.G1720A), which leads to an arginine-to-histidine exchange [UPQ41334.1:p.Arg305His]. The Ultramel animal was homozygous for this variant, termed hereafter R305H.”

Fig. 4 The part of legend in (B) which states "Ultramels heterozygous for R305H...when heterozygous." should be moved in to the main Results text around L333. And it should be more explained. Were all the amplicons detected in the 2 heterozygous animals? Can you show the PCR picture of the heterozygote (in Fig. S1 would be enough)?

Thanks for encouraging us to add more detail related to the Ultramels heterozygous for R305H. We have revised the main text to explain why the deletion cannot be detected in heterozygotes. The text reads,

“Loss of these coding regions was specific to Ultramels lacking the R305H variant and did not occur in any of the Non-Ultramels, nor in the Ultramels homozygous or heterozygous for R305H (Fig 4B). A simple explanation of this pattern is that the Ultramels lacking R305H were homozygous for a deletion spanning coding regions 6 and 7 of TYRP1. The two Ultramels heterozygous for R305H were presumed to be compound heterozygous for this putative deletion, although we were not able to confirm this heterozygosity because the putative deletion extended into a discontinuity in the Burmese python reference genome (and therefore could not be detected in heterozygotes).”

The genotypes of the two heterozygous animals are provided in S6 Table, which indicates presence/absence of a PCR product spanning coding regions 6 and 7 of TYRP1. Presence of this PCR product is sufficient to conclude that these animals have an intact copy of coding regions 6 and 7 on at least one chromosome. We have added an explanation of the assay, as follows,

“These primers reside within the putative deletion and therefore cannot distinguish between animals lacking the putative deletion and animals heterozygous for it. Presence of a PCR product in this assay indicates that TYRP1 coding regions 6 and 7 are present on at least one chromosome. Absence of a PCR product in this assay indicates that TYRP1 coding regions 6 and 7 are absent on both chromosomes. We were unable to design a PCR assay to detect the putative deletion in heterozygotes because the putative deletion extends into a discontinuity in the Burmese python reference genome. Presence or absence of a PCR product in this assay is provided in S6 Table.”

L337-339 remove the parentheses

Thank you for the suggestion. We have revised the text accordingly.

L386 "slicing" should read splicing

Thank you for finding this mistake. We have fixed it.

L447 Current state-of-the-art methods (such as targeted high-throughput sequencing, whole-genome sequencing, or long-read technologies) should be mentioned here as an obvious solution to missing reference and/or structural variants. See (cite) e.g. <https://www.mdpi.com/2073-4425/10/5/386/htm> for an example of a coat color gene variant identification without correct reference sequence.

We agree that long-read technologies provide the most straightforward avenue for improving genome assemblies in pythons and identifying structural variants. We have revised the text to include this point. The text reads,

“As genomic resources for Burmese python and ball python expand, ideally with the use long-read technologies to improve genome assemblies and identify structural variants, we expect to identify a putatively causative variant on the TYRAlbino haplotype and to map the breakpoints of the putative deletion in TYRP1.”

L454 "strip" should be stripes

Thank you for finding this mistake. We have fixed it.

L485 "supports" at the end of sentence should probably be deleted

Thank you for finding this mistake. We have fixed it.

L487 How many were "Toffee" or "Candy"?

We have revised the text accordingly. It now reads,

“The Albinos were composed of 36 animals described as AlbAlbino homozygotes, three animals described as AlbCandy homozygotes, one animal described as an AlbToffee homozygote, nine animals described as AlbAlbino / AlbCandy compound homozygotes, and one animal described as an AlbAlbino / AlbToffee compound homozygote.”

L525 Please, specify the software used. Does TBLASTN refer to the NIH Translated BLAST tool? Citation should be provided, as well as clarification, if default parameters were used for the search or if anything was adjusted.

We have added a brief explanation of TBLASTN, as well as citations for it. We have also specified that TBLASTN searches were performed using default parameters. This text reads,

“To curate new gene annotations, we performed TBLASTN [110,111] searches of the Burmese python genome (Python_molurus_bivittatus-5.0.2) using protein sequences from anole lizard or corn snake as the query. TBLASTN is a search tool that aligns a query protein sequence all six reading frames of a nucleotide database. TBLASTN searches were performed using default parameters.”
